# Tonic Shock Induces Detachment of *Giardia lamblia*

**DOI:** 10.1371/journal.pntd.0000169

**Published:** 2008-02-13

**Authors:** Wendy R. Hansen, Daniel A. Fletcher

**Affiliations:** 1 Biophysics Graduate Group, University of California, Berkeley, California, United States of America; 2 Department of Bioengineering, University of California, Berkeley, California, United States of America; Bose Institute, India

## Abstract

**Background:**

The parasite *Giardia lamblia* must remain attached to the host small intestine in order to proliferate and subsequently cause disease. However, little is known about the factors that may cause detachment *in vivo*, such as changes in the aqueous environment. Osmolality within the proximal small intestine can vary by nearly an order of magnitude between host fed and fasted states, while pH can vary by several orders of magnitude. *Giardia* cells are known to regulate their volume when exposed to changes in osmolality, but the short-timescale effects of osmolality and pH on parasite attachment are not known.

**Methodology and Principal Findings:**

We used a closed flow chamber assay to test the effects of rapid changes in media osmolality, tonicity, and pH on *Giardia* attachment to both glass and C2_Bbe_-1 intestinal cell monolayer surfaces. We found that *Giardia* detach from both surfaces in a tonicity-dependent manner, where tonicity is the effective osmolality experienced by the cell. Detachment occurs with a characteristic time constant of 25 seconds (SD = 10 sec, *n* = 17) in both hypo- and hypertonic media but is otherwise insensitive to physiologically relevant changes in media composition and pH. Interestingly, cells that remain attached are able to adapt to moderate changes in tonicity. By exposing cells to a timed pattern of tonicity variations and adjustment periods, we found that it is possible to maximize the tonicity change experienced by the cells, overcoming the adaptive response and resulting in extensive detachment.

**Conclusions and Significance:**

These results, conducted with human-infecting *Giardia* on human intestinal epithelial monolayers, highlight the ability of *Giardia* to adapt to the changing intestinal environment and suggest new possibilities for treatment of giardiasis by manipulation of tonicity in the intestinal lumen.

## Author Summary

The single-celled organism *Giardia lamblia* colonizes the small intestine of a wide variety of hosts, including humans. Giardiasis infections can cause severe gastrointestinal symptoms and pose a major health concern in the developing world. *Giardia* are known to attach robustly to a variety of surfaces, but the conditions that influence this attachment are not known. In this study, we examined the behavior of attached *Giardia* parasites exposed to rapid changes in solution properties, like those *Giardia* might encounter in the intestine. After systematically varying media concentration and composition, we found that only one solution property caused rapid detachment of *Giardia* cells: tonicity, which is a measure of the total concentration of solutes in the solution that are unable to pass through a semi-permeable membrane (here, the cell membrane of *Giardia*). We found similar results for *Giardia* initially attached to monolayers of intestinal cells. *Giardia* cells remaining attached after a change in tonicity are able to adapt to the change, highlighting the general ability of this organism to weather normal changes in the intestinal environment. We propose that *Giardia*'s susceptibility to large changes in tonicity could be explored as a possible new route for treatment of giardiasis.

## Introduction

The parasitic protozoan *Giardia lamblia* is a major cause of diarrheal illness and infects millions of people per year, primarily via waterborne outbreaks in developed countries and long-term water contamination in developing countries [1],[2]. *Giardia* trophozoites infect many hosts, including humans, preferentially colonizing the proximal small intestine, which is sparsely populated by other microbes in comparison to the rest of the intestinal tract [3],[4]. Symptoms of giardiasis vary among patients, ranging from asymptomatic infections to malabsorption and severe chronic diarrhea. The health impacts of giardiasis can be severe, as chronic infection or reinfection may cause malnutrition and growth retardation [1],[5]. Giardiasis is generally treated with metronidazole or other nitroimidazoles, which target the parasite's anaerobic metabolism [6], as well as with furazolidone or quinacrine. However, these chemotherapeutic agents are not always available, fully effective, or tolerated [6],[7].

While the exact mechanism of virulence is not well understood [5], attachment of *Giardia* to the small intestine wall is a requirement, since unattached cells would simply pass through the intestine during peristalsis. Thus attachment, also a requirement for cell proliferation in the host, can be considered a virulence factor in giardiasis. Trophozoites in culture have been observed to attach and detach in less than one second [8],[9], and they are able to attach to a variety of surfaces, both biological and inert. Attachment itself has been studied extensively (reviewed in [8],[10]), but the contributing environmental factors remain unclear, as does an understanding of how *Giardia* are able to remain attached (or reattach when necessary) in the highly variable environment of the small intestinal lumen.

In addition to resisting shear stresses resulting from peristaltic flow, *Giardia* must withstand variations in the osmolality and pH of the contents of the small intestine resulting from normal feeding behavior of the host. Osmolality is defined as the total concentration of solutes per kilogram of solvent. Strictly speaking, for a semi-permeable membrane that passes some solutes, such as a cell membrane, the most relevant measure is tonicity, or the concentration of membrane-impermeant solutes per kilogram of solvent. Osmolality in most organisms is tightly regulated, and in humans, serum osmolality is held at 290 mOsm/kg. In the upper digestive tract, however, osmolality and pH can vary considerably from fed to fasted states. The largest variability is in the antrum and the duodenum, where osmolality has been found to range from approximately 30% to more than 200% of serum levels in fasted and fed states [11], and pH fluctuations below pH 5 and above pH 7.5 have been observed [11],[12]. Both pH and osmolality stabilize somewhat in more distal portions of the small intestine [13]–[15].

**Figure 1:**
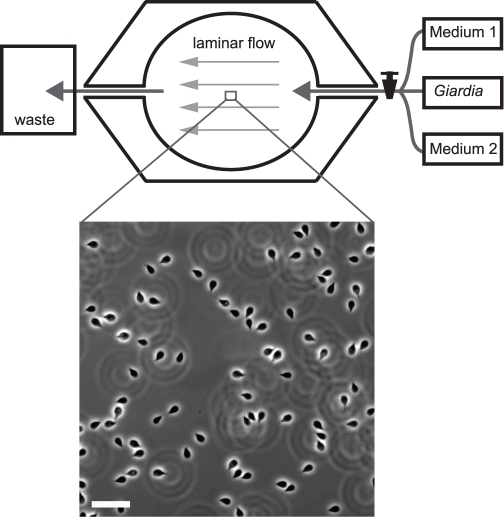
Schematic of the closed laminar flow chamber setup used to test attachment of *Giardia* under varying media conditions. *Giardia*, test medium, and control medium were flowed in separately at controlled flow rates. The micrograph shows a 20X phase image of a representative population of *Giardia* attached to glass. Attachment levels are quantified by counting the attached cells in the population during media changes. Scale bar = 50 µm.

To study the effect of osmolality and pH changes on *Giardia lamblia* attachment, we developed a flow chamber assay to monitor cell attachment during changes in media concentration and composition (Figure 1). The closed chamber prevented exposure to excessive oxygen, as *Giardia* are microaerophilic, and it allowed us to monitor attachment to both glass surfaces and intestinal epithelial monolayers under controlled flow rates, while quickly changing media. We tracked changes in the number of attached cells during the experiments to quantify effects on attachment. Our results show that *Giardia* rapidly detached from both glass and enterocyte monolayer surfaces when osmolality was altered from standard growth medium conditions. We found that the fraction of detached cells increased with the magnitude of osmolality change. Experiments with iso-osmotic solutions containing solutes that can and cannot pass through the cell membrane (non-isotonic and isotonic, respectively) indicate that tonicity is the critical factor causing detachment. Interestingly, we observed that *Giardia* are able to adapt to moderate changes in tonicity, indicated by the dependence of detachment on the change in tonicity rather than the absolute tonicity. Based on *Giardia*'s adaptation to environments of different tonicities, we were able to vary solution tonicity periodically in a way that forced extensive detachment of *Giardia* from both glass and epithelial monolayers.

## Methods

### Culture of *Giardia* and C2_BBe_-1 cells

*Giardia lamblia* WBC6-strain trophozoites were cultured in 15-mL polystyrene tubes at 37C in growth medium [16] supplemented with bovine bile (Sigma Aldrich, St. Louis, MO), adult bovine serum (Biosource International, Camarillo, CA) and penicillin/streptomycin/Fungizone (Cambrex Bio Science, Baltimore, MD). Cultures were passaged three times per week. For experiments, cells were grown to confluency and detached with a 20-minute cold shock at 4C.

Two experiments were performed to test the effect of hypo- and hyperosmotic media conditions on growth. First, to test cell viability after prolonged exposure to test media, *Giardia* were passaged as usual into growth media containing approximately 20–200% normal solute concentration, or osmolality ranging from 62 to 625 mOsm/kg. Second, to test viability of cells after brief exposure to these media, cells grown in normal growth medium were detached by cold shock, centrifuged at 1500 *g*, and resuspended in test media for three minutes. Cells were then centrifuged again and resuspended in normal growth medium. For both viability and growth controls, cells were counted using a hemacytometer after 24 hours of growth. Cells were also regularly monitored in tubes for confluency levels.

C2_BBe_-1 cells, which are brush border expressing clones of Caco-2 intestinal epithelial cells [17], were obtained from the American Type Culture Collection (ATCC) and cultured at 37C in Dulbecco's modified Eagle's medium (DMEM, ATCC 30-2002) with 10% fetal bovine serum (Mediatech, Herndon, VA), penicillin/streptomycin, and 0.01 mg/mL human apo-transferrin (Sigma, St. Louis, MO). Culture medium was changed three times per week and cells were passaged at 80−90% confluency, approximately every six days. For experiments, C2_BBe_-1 cell monolayers were grown on glass coverslips for at least three weeks beyond confluency to allow complete differentiation and microvillus brush border growth [17],[18]. C2_BBe_-1 cells were not used beyond passage 68 due to possible brush border instability [17].

### Flow chamber experiments

#### Giardia *on glass*

Chilled *Giardia* cells were centrifuged at 1500 *g* for five minutes, and the pellet was resuspended in chilled medium and kept on ice. A sealed flow chamber (RC-30, Harvard Apparatus, Holliston, MA) was washed with medium and maintained at 37C with a temperature controller (TC342B, Warner Instruments, Hamden, CT). *Giardia* cells were then flowed in and allowed to attach to the glass surface of the flow chamber for five minutes (Figure 1). Next, time-lapse videos of cell attachment under 20X phase illumination on an inverted microscope (Axiovert 200, Carl Zeiss MicroImaging, Thornwood, NY) were acquired with a Cascade II CCD camera (Photometrics, Tucson, AZ) at 1 frame/second. Flow rates in the chamber were controlled using syringe pumps (PHD 22/2000 and Pump 11, Harvard Apparatus). The chamber was first flushed of unattached cells by flowing in unmodified growth medium at 0.3 mL/min for three minutes at the beginning of each time-lapse. Then experimental medium was flowed in at 0.3 mL/min. Between experiments, the chamber was flushed with deionized H_2_O to detach remaining *Giardia* cells in order to avoid selection of more strongly adherent cells.

In most cases, test media formulations were based on normal *Giardia* growth medium. Variations in osmolality were established by preparation of media with additional or less water as necessary. High and low pH isotonic media were prepared by slight dilution of HEPES-based medium with water, followed by adjustment of pH with NaOH or HCl, then by a final adjustment of osmolality with sucrose. Medium osmolality was measured with a vapor pressure osmometer (Wescor, Logan, UT) and confirmed for selected samples with a freezing point depression osmometer (Precision Systems, Natick, MA).

Cell numbers were counted using MetaMorph analysis software (Molecular Devices Corporation, Sunnyvale, CA). All other data analysis was done with Igor Pro (WaveMetrics, Lake Oswego, OR) except for chi-square statistical analysis, which was done with SPSS software (SPSS Inc., Chicago, IL). Cell counts were averaged for the 10 frames immediately prior to exposure to test media (once unattached cells were flushed from the chamber); this number was used to normalize cell counts throughout the experiment. All attachment numbers are reported as the normalized cell attachment index. Significance levels, established using chi-square analysis, were in comparison to attachment in unmodified growth medium. Significant differences (p<0.05) are indicated by asterisks in figures. Error bars indicate standard deviation for experiments with more than one trial.

To study the behavior of *Giardia*'s flagella during tonicity-induced detachment, high-resolution imaging experiments were performed with diluted growth medium, using 100X phase contrast video microscopy on the microscope described above. Images were continuously acquired during media exchange to capture the moment of detachment at high magnification.

#### Giardia *on C2_BBe_-1 monolayers*

C2_BBe_-1 cells were seeded onto glass coverslips and grown as described above. The flow chamber was assembled with monolayer-coated coverslips as the top surface of the chamber and immediately flushed with warmed medium.

*Giardia* cells were incubated for 20 minutes at 37C with 10 µM CellTracker Orange CMTMR fluorescent dye (Invitrogen Corporation, Carlsbad, CA) in 0.1% DMSO. The cells were then chilled for 20 minutes, centrifuged and washed with growth medium three times, and flowed into the assembled flow chamber. The chamber was inverted for 10 minutes to encourage cell attachment to the C2_BBe_-1 monolayer (top coverslip). For experiments, control medium was flowed through the chamber for three minutes, then five images were taken in both phase and fluorescence illumination. Test medium was then flowed through the chamber for another three minutes and a second set of images was taken. Images were not continuously acquired in order to minimize photobleaching of the *Giardia* cells. Most experiments were limited to 35 minutes, as longer experiments sometimes disrupted the C2_BBe_-1 monolayers. The fluorescent images were processed with a top-hat filter and thresholded to identify and count *Giardia* cells, and counts were compared with the phase images for accuracy.

For experiments with multiple tonicity swings, hypertonic and hypotonic media were made from a dry mixture (Colyte, Schwarz Pharma, Mequon, WI) of poly(ethylene glycol) (PEG-3350, 86.25% w/w), NaCl (2.10%), KCl (1.07%), NaHCO_3_ (2.42%), and Na_2_SO_4_ (8.16%). This mixture was reconstituted with sufficient deionized H_2_O to create the desired tonicity levels.

### Scanning electron microscopy

To confirm brush border expression, fully differentiated C2_BBe_-1 cell monolayers grown on pieces of glass coverslips were gently washed with serum-free DMEM, fixed in 2% glutaraldehyde in 0.1 M Na cacodylate for 1.5 hours, rinsed with Na cacodylate buffer, post-fixed with 1% osmium tetroxide in 0.1 M Na cacodylate buffer for one hour, rinsed again, and dehydrated in an ethanol series.

For coincubated samples, *Giardia* cells were centrifuged at 1500 *g* for five minutes and the pellet was resuspended in cysteine-buffered DMEM and added to tissue culture dishes with C2_BBe_-1 monolayers on glass coverslip pieces. *Giardia* were allowed to attach to the C2_BBe_-1 monolayer for 45 minutes, then both were fixed together using the procedure previously described.

All samples were critical point dried in an AutoSamdri 815 drier (Tousimis Research, Rockville, MD), mounted to stubs using carbon tape, sputter coated with 2.4 nm of platinum using a MED020 sputter coater (Bal-Tec AG, Liechtenstein) and viewed with an S-5000 scanning electron microscope (Hitachi High Technologies America, Pleasanton, CA).

## Results

### Variation of media osmolality and pH

To determine the effect of solution properties on attachment, we exposed attached *Giardia* trophozoites to changes in osmolality, tonicity, and pH using a flow chamber assay. Media variations of approximately 60 to 620 mOsm/kg and pH 5 to pH 8 were selected to span the range measured in the human proximal small intestine [11]–[15]. We limited bulk flow rate to 0.3 mL/min, a rate that flushed unattached cells from the chamber but did not perturb attached cells. At significantly higher flow rates (approximately 3 mL/min and higher), cells first oriented upstream into the flow and then were forced to detach by the shear stress of the flow (data not shown). We found that when detachment of cells occurred, the response was very rapid, and detachment was complete within less than two minutes (including media exchange time) for a given population, so we limited test media presentation to three minutes unless otherwise noted. Longer experiments (up to 60 min) did not result in significant additional detachment unless the medium composition was not suitable for cell growth (e.g. sucrose solutions or media lacking cysteine; data not shown).

**Figure 2:**
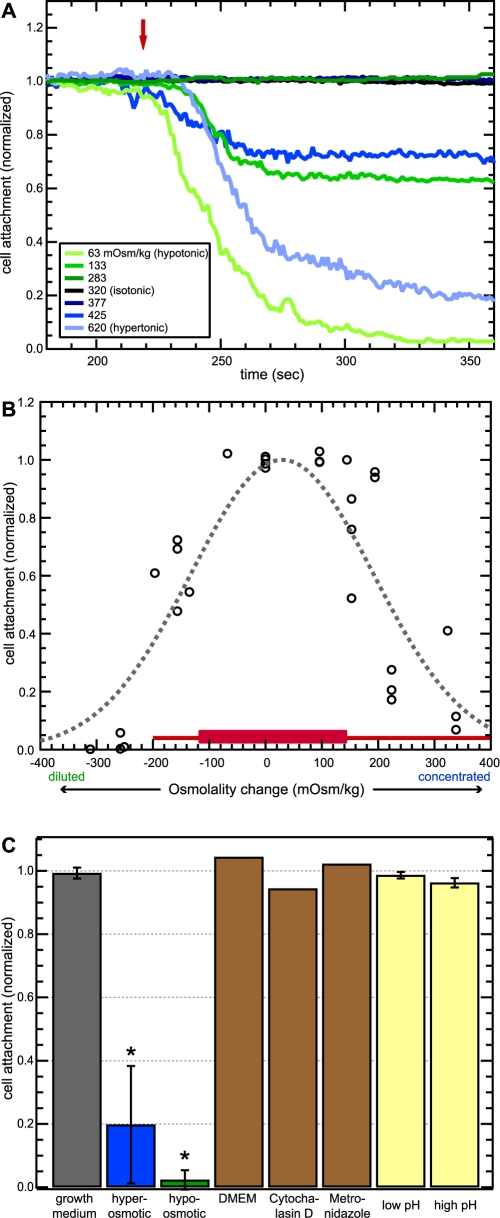
Detachment of *Giardia* from glass. (A) Detachment from glass during three-minute exposure to growth media of varying osmolality. Traces are normalized counts of attached cells averaged over 3−4 independent experiments for each osmolality. Red arrow marks the time where test media reached cells. Shifting the medium osmolality either progressively higher (from dark blue to light blue traces) or progressively lower (from dark green to light green traces) caused cells to detach in correspondingly greater numbers. (B) Cell attachment levels after three-minute exposure to growth media of varying osmolality for all osmolalities tested. Data fit a Gaussian profile (dashed line). For comparison, the red line indicates the full range of osmolality measured in the human duodenum of fed and fasted subjects and the red box indicates range of mean values [11]. (C) Mean normalized attachment levels in control experiments. *Giardia* did not show significant short-timescale detachment in the presence of *Giardia* growth medium, C2_BBe_-1 growth medium (DMEM), growth media with cytochalasin D (0.1 mM) or metronidazole (30 µg/mL), or in HEPES-based media with elevated pH (pH = 8.03) or low pH (pH = 4.94). Asterisks indicate significant difference from attachment levels in unmodified growth medium (p<0.000005 for both). Error bars indicate standard deviation.

As a first test of osmotic stress response, we exposed cells to a range of dilutions and concentrations of standard complete growth medium. We monitored cell attachment numbers during the three-minute exposure period and found no significant effect on attachment for osmolality shifts smaller than ∼±30% from the baseline medium osmolality of 300−330 mOsm/kg. Above ∼30% shifts in osmolality (osmolality below ∼230 mOsm/kg or above ∼430 mOsm/kg) both hypo- and hyperosmotic solutions resulted in rapid detachment of a statistically significant fraction of attached cells (Figure 2A,B). Detachment began within 10 seconds of exposure to test media and depended on the magnitude of osmotic change. In extremely hypo-osmotic media (62.5 mOsm/kg or lower), 97.7% (SD 3.03%, *n* = 3) of cells detached from the glass surface, while in highly hyperosmotic media (620 mOsm/kg or higher), 80.2% (SD = 18.5%, *n* = 3) of cells detached. Chi-square analysis found these to be significantly different (p<10^−6^ for both). For those experiments in which cells detached, they did so with a time constant τ = 25 seconds (SD = 10 sec, *n* = 17) when fitted to an exponential decay function.

Control experiments showed that *Giardia* did not detach from glass on the three-minute timescale of our experiments in the presence of unmodified *Giardia* growth medium or when exposed to the iso-osmotic Dulbecco's Modified Eagle's medium (DMEM) used for culturing C2_BBe_-1 intestinal epithelial cells (Figure 2C). In previous studies, the actin depolymerizing agent cytochalasin D was associated with detachment after 24 hours of exposure [19]–[21]. Here, iso-osmotic addition of cytochalasin D (0.1 mM) to *Giardia* growth medium had no significant effect on attachment after three minutes of exposure (Figure 2C). Finally, metronidazole (30 µg/mL), the current drug of choice for treatment of giardiasis [6], also did not cause *Giardia* to detach from glass on the three-minute timescale (Figure 2C).

To test whether exposure to growth media of different osmolality caused detachment by killing cells, we conducted additional control experiments. We found that *Giardia* populations briefly exposed (three minutes) to hypo- or hyperosmotic growth media and then returned to normal growth medium had normal morphology and behavior under the microscope and grew at normal rates (Figure S1A). This suggests that short-timescale osmolality shifts did not induce detachment by killing cells. However, cells incubated for 24 hours in hypo- or hypertonic media, though unaffected by small changes in osmolality, grew more slowly under moderate osmotic stress, and were not viable in the most extreme hypo- and hypertonic media tested (Figure S1B).

We also tested the effect of pH on detachment because it is known to vary considerably in the duodenum [11],[15] and somewhat in the jejunum [13],[14]. After three minutes, low pH (pH = 4.94) medium had attachment levels of 98.7% (SD = 1.1%, *n* = 3), while high pH (pH = 8.03) medium had attachment levels of 96.3% (SD = 1.4%, *n* = 3, Figure 2C). Even though these physiologically relevant changes in pH did not result in significant detachment, media pH during the time course of other experiments was held relatively constant.

### Adaptation to osmolality changes

**Figure 3:**
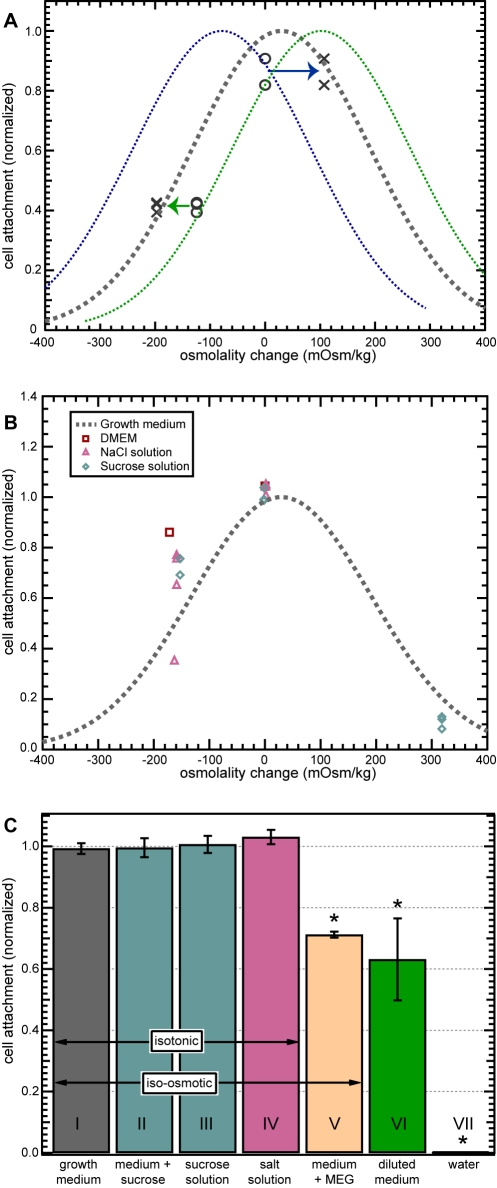
Detachment under changes in solution composition, osmolality, and tonicity. (A) Cells initially incubated in growth media of a slightly higher (green) or lower (blue) osmolality adjusted to the shifted osmolality of the incubation medium. Subsequent exposure to test media of lower and higher osmolality produced detachment in response to the magnitude of the osmotic shift from the new osmolality set point, not the original osmolality. Arrows indicate shift between the observed detachment behavior (circles) and the detachment behavior that would be expected if cells did not adjust (crosses). (B) Cell attachment versus osmotic change in all media used. Dashed line is the fit for attachment results in growth media of varying osmolality (Figure 2B). Similar osmolality-dependent results were found for exposure to DMEM growth medium (squares) and for brief exposure to pure NaCl (triangles) or sucrose (diamonds) solutions. (C) Isotonicity experiments. Bars represent the mean normalized cell attachment at the end of the three-minute experiment. In the presence of growth medium alone, cells did not detach. In medium diluted by half with water, 29% of cells detached (p = 0.00001), while all cells exposed to pure water detached (p<0.000005). This effect can be prevented by restoring the diluted medium to its original tonicity with sucrose, or by substituting a pure, isotonic sucrose or NaCl solution. The effect is not prevented by restoring medium osmolality with cell-permeant mono(ethylene glycol) (MEG), which is iso-osmotic but not isotonic (p = 0.0095). Error bars indicate standard deviation.

We next explored whether *Giardia* detachment behavior was dependent on the absolute osmolality of the solution or on the magnitude of osmotic change from one solution to the next. That is, would *Giardia* still react similarly to changes in solution osmolality like those documented above if they started out in a medium of different osmolality than normal growth medium? To this end, *Giardia* were initially incubated for 10 minutes in growth media of slightly elevated or lowered osmolality (by +79 mOsm/kg and −107 mOsm/kg, respectively). Cells were then exposed to a moderate osmotic shift in the opposite direction. For the cells incubated in +79 mOsm/kg medium, the test medium osmolality was 182 mOsm/kg, or 124 mOsm/kg below the incubation medium and 197 mOsm/kg below normal medium tonicity. For cells incubated in the –107 mOsm/kg medium, test medium osmolality was a normal 308 mOsm/kg. In both cases, when cells were subsequently exposed to these osmolality shifts, which were of an absolute osmolality that would not normally result in much detachment, a statistically significant percentage of cells detached. The amount of detachment depended on the osmotic difference between the incubation medium and test solution instead of on the absolute value of the incubation medium and test solution osmolality. That is, cells appeared to adapt to the osmolality of their incubation medium. As a result, detachment for the given osmolality change is similar to that for cells initially incubated in normal medium (300−330 mOsm/kg) and exposed to the same magnitude of osmotic change (Figure 3A).

### Pure, isotonic solutions prevent detachment

To examine whether *Giardia* detachment behavior depended on the specific composition of the medium, we tested several different solutions with a range of osmolalities. Surprisingly, the detachment trends observed in hypo- and hyperosmotic growth media (Figure 3B, dashed line) were also seen for solutions that do not support cell growth or survival (Figure 3B). Pure NaCl and pure sucrose solutions induced no detachment of *Giardia* cells when the solution osmolarity matched that of growth medium. Detachment levels in both pure NaCl and pure sucrose solutions of varying osmolality were found to match results for those in growth medium, indicating that cell detachment is not an immediate response to nutrient deprivation. However, cells left in sucrose for a longer time period gradually began to detach after approximately 10 minutes of exposure, and long-timescale exposure (>60 minutes) resulted in cell death.

The detachment-inducing effect of reduced-osmolality growth medium can be prevented by restoring diluted medium to its original osmolality with the addition of solutes, e.g. sucrose, before exposing cells to it (Figure 3C). In this experiment, we diluted medium by 150 mOsm/kg, causing 36.9% of cells to detach after three minutes (Figure 3C, column VI, p = 0.00001) in comparison to undiluted medium (column I). However, supplementing diluted medium with a sufficient amount of sucrose to restore its original osmolality resulted in 99.6% (SD = 3.1, *n* = 3, column II) of cells remaining attached. Direct substitution of an iso-osmotic solution of either pure sucrose (column III) or pure NaCl (column IV) for growth medium also prevented detachment, with attachment at 100.6% (SD = 2.8%, *n* = 3) and 103.1% (SD = 2.4%, *n* = 3) of control levels, respectively. Thus, the observed detachment is not due to the absence of specific osmolytes in the test medium.

However, restoration of medium osmolality from a dilute 162 mOsm/kg to 324 mOsm/kg with cell-permeant mono(ethylene glycol), or MEG, resulted in detachment levels comparable to those in the diluted growth medium, with 28.8% of cells detaching (column V, p = 0.009). Thus, changes in media tonicity, not osmolality, cause detachment, where tonicity is the effective osmolality due to cell-impermeant osmolytes. This effect appears to be independent of medium composition for all media tested. We refer to the sudden detachment of *Giardia* in response to tonicity changes as “tonic shock”.

### *In vitro* intestinal model

**Figure 4:**
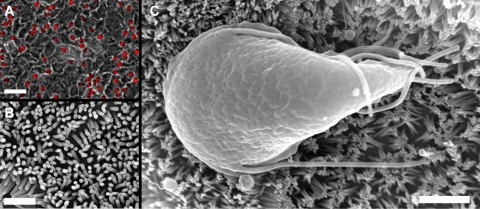
C2_BBe_-1 monolayers as a model for studying *Giardia* attachment to the intestinal epithelium. (A) Fluorescent image of *Giardia* (red) attached to a C2_BBe_-1 cell monolayer, overlaid on a 20X phase image. Scale bar = 50 µm. (B) Scanning electron micrograph of brush border-expressing C2_BBe_-1 monolayer cells grown on glass showing dense microvilli. Scale bar = 1 µm. (C) Scanning electron micrograph of *Giardia* attached to a C2_BBe_-1 cell monolayer. Scale bar = 2 µm.

To test whether the observed tonicity-dependent detachment was specific to glass substrates, we repeated key experiments with *Giardia* attached to a monolayer of C2_BBe_-1 cells, which are a brush border-expressing sub-clone of Caco-2 cells and are well established as a model for the intestinal epithelium [17],[18],[22]. Fluorescently-labeled *Giardia* attached readily to these monolayers (Figure 4A) and scanning electron microscopy confirmed that our method produced cell monolayers with dense microvilli (Figure 4B) to which *Giardia* can attach (Figure 4C).

**Figure 5:**
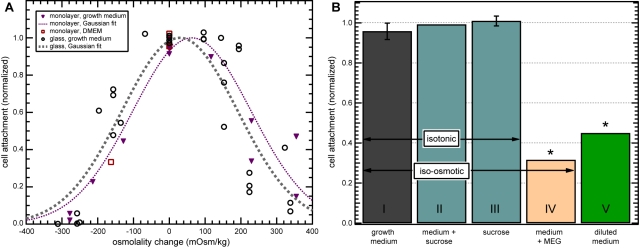
Detachment of *Giardia* from intestinal epithelial monolayers. (A) *Giardia* detachment from C2_BBe_-1 monolayers in hypo- and hyperosmotic growth media (purple triangles) and a Gaussian fit (thin dotted line). Data from experiments on glass (Fig. 2B) are included for reference (black circles, thick dotted line). (B) Isotonicity experiments on monolayers. Results are similar to those for *Giardia* attached to glass (Fig. 3C), with MEG-supplemented medium failing to prevent detachment (p<0.000005), as did simple diluted medium (p = 0.0007). Error bars indicate standard deviation.

*Giardia* attached to intestinal monolayers exhibited tonicity-dependent detachment behavior remarkably similar to that of *Giardia* attached to glass. Cells detached from the monolayers in both hypo- and hypertonic media, and the number of detached cells depended on the magnitude of tonic change (Figure 5A, triangles). This was observed for both experiments conducted in *Giardia* growth medium and C2_BBe_-1 DMEM growth medium (Figure 5A).

As in the osmolality restoration experiments for *Giardia* on glass (Figure 3C), the detachment response on C2_BBe_-1 monolayers was prevented only by isotonic media. In undiluted medium at 335 mOsm/kg, 95.8% (SD = 4.12%, *n* = 3) of cells remained attached after experiments (Figure 5B, column I), while medium diluted to 206 mOsm/kg (column V) dropped attachment levels to 44.9% (p = 0.0007). When this diluted medium was restored to its original osmolality with sucrose (column II), 99.2% of cells remained attached. Similarly, an iso-osmotic pure sucrose solution (column III) resulted in 102.6% of initial attachment (100.9% and 104.4%, *n* = 2). Finally, medium diluted by 140 mOsm/kg and then supplemented to iso-osmotic levels with cell-permeant MEG (column IV) failed to prevent detachment, with only 31.5% of cells remaining attached after exposure (p = 0.00005).

### Tonicity manipulation to force detachment

As previously described, *Giardia* cells that remain attached after exposure to changes in medium tonicity appear to adapt to the new tonicity (Figure 3A). We hypothesized that if these cells were truly adjusting to a different medium tonicity, this adaptation would render them sensitive to a small deviation from the original medium tonicity that was insufficient to cause detachment before adaptation. For example, cells that had previously adapted to a slightly hypertonic medium would be more sensitive to a small hypotonic challenge than if they did not adapt. The small hypotonic challenge, in turn, would make the cells more sensitive to a subsequent exposure to hypertonic medium if the cells adapted to the hypotonic medium.

To test this idea, we designed an experiment in which *Giardia* attached to C2_BBe_-1 monolayers were exposed to hypertonic medium flow for three minutes, then left to adjust to the new tonicity for five minutes, then challenged with hypotonic medium flow for three minutes, then allowed to adjust again for five minutes. The tonicity fluctuation pattern was then repeated, alternating between hypo- and hypertonic values that were either ±100 mOsm/kg or ±150 mOsm/kg from the original growth medium tonicity. These tonicity values were selected to correspond with values producing only mild detachment in previous experiments (Figure 2), which are also consistent with osmolality fluctuations measured in the human small intestine [11]. For these experiments we used a PEG/electrolyte mixture as the medium base.

**Figure 6:**
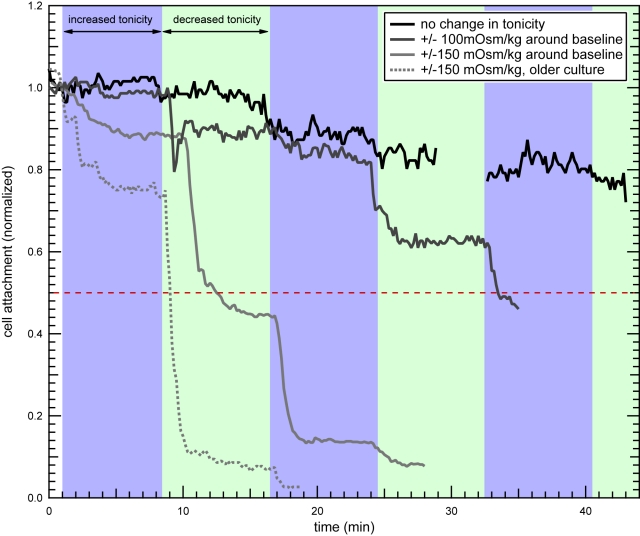
Enhanced detachment with controlled tonicity swings. *Giardia* attached to C2_BBe_-1 monolayers were exposed to hypertonic and hypotonic growth media (blue and light green panels, respectively) in a timed pattern selected to exploit cells' adjustment to tonicity changes. Nearly all cells exposed to swings of ±150 mOsm/kg followed by adjustment periods detached over the course of the experiments (light gray trace), and over half (red dashed line) of cells exposed to swings of ±100 mOsm/kg detached (gray trace). Cells cultured for 72 hours instead of 48 hours prior to experiments appeared to detach more readily (dotted line). Traces are the averaged attachment index for multiple experiments (*n = *2 for ±100 mOsm/kg, *n = *5 for 48-hour ±150 mOsm/kg, *n = *3 for 72-hour ±150 mOsm/kg).

Figure 6 shows that cells remaining attached do indeed adapt to the tonicity of the surrounding solution and that tonicity manipulations can force a larger fraction of cells to detach. In hyper- and hypotonic media with swings ±150 mOsm/kg from standard medium tonicity, cells detached in a stair-step pattern, and average attachment was reduced to 13.6% (SD = 4.8%, *n* = 5) after 2 periods each of hypotonic and hypertonic medium exposure (Figure 6, Video S1). In media ranging ±100 mOsm/kg from standard tonicity, detachment was more gradual, but over half of initially attached cells had detached by the third exposure to hypertonic medium (44.7% and 47.4% attachment, *n* = 2). Cells incubated for an additional 24 hours prior to experiments appeared to detach more readily (Figure 6, dotted line), with average attachment dropping to 8.4% after one period each of hyper- and hypotonic medium exposure (SD = 7.6%, *n = *3). In comparison, the constant tonicity control showed 81.6% attachment at this time point. Some detachment is expected in long timescale experiments since cells detaching for any reason are swept away by the flow. However, this gradual decline in cell numbers is clearly different from the stair-step pattern seen during the periodic tonicity swings.

## Discussion

*Giardia* trophozoites rely on attachment to stay within the host and must accommodate a large range of perturbations in flow and chyme composition. In these experiments, we used live cell microscopy to monitor immediate detachment events upon exposure to test media in a flow chamber. Short-timescale detachment of *Giardia* has not been examined in the numerous studies of forced detachment, as most assays measure population attachment levels after a period of 2−24 hours incubation with a given drug or medium composition (e.g. [19]–[21],[23]). Such studies have shown that *Giardia* attachment and survival rates are inhibited by changes in pH and osmolality [23], but the long incubation prior to quantification of attachment makes it very difficult to separate direct effects on attachment from an overall effect on cell viability. Our results demonstrate that not only are *Giardia* susceptible to tonic shock, but that the response is rapid, with a detachment time constant of 25 seconds. This is consistent with the extremely rapid attachment and detachment behavior of trophozoites noted in the literature [8],[9]. Notably, remaining cells appear to adapt to the new tonicity and are not susceptible to further detachment unless subjected to a second tonic shock.

It is unclear whether the tonicity-dependent detachment we observe is because tonic changes directly affect *Giardia*'s mechanism of attachment or whether detachment is a secondary result, perhaps due to observed osmotic shrinking and swelling of the cell. Attachment is associated with a unique microtubule-based structure called the ventral disk, which is located on the underside of the cell. Other studies have hypothesized several methods of attachment, including specific binding by lectins to the surface of intestinal cells (reviewed in [10]). High resolution (100X) phase contrast video microscopy of attached *Giardia* under hypotonic shock (−196 mOsm/kg) showed that detachment events are non-catastrophic, with affected cells simply “falling off” the glass surface (Video S2). Beating of the ventral flagella was sinusoidal as previously observed [24], even during detachment, and cell morphology appeared unchanged.

Given *Giardia*'s ability to adhere to glass and other inert surfaces and the observation that detachment forces are unaffected by surface chemistry [25], a pressure-based mechanism of attachment [26]–[28] appears most likely. Yet the major question of how pressure is generated remains open. We propose that the detachment behavior observed in this study is consistent with an osmotic, pressure-based mechanism of attachment in which cells create an osmotic pressure differential to generate attachment force. Osmolyte leak rate or a compromised seal around the ventral disk would be the primary modes of attachment failure under tonic shock. Although this model is consistent with our data, we know of no example in the literature of a single-celled organism that generates an osmotic gradient for attachment.

Previous studies of the regulatory volume decrease of unattached cells showed that *Giardia* cells release alanine and potassium to decrease intracellular osmolality upon exposure to hypotonic media [29]–[31]. Regulatory volume changes are present in most eukaryotes and aid the cell in adjusting to a range of medium tonicities; if *Giardia* do indeed use an osmotic method of attaching to surfaces, the correspondence between volume changes and attachment in *Giardia* raises the question of whether *Giardia* evolved their robust attachment mechanism from an ancestrally conserved volume regulation mechanism or whether these mechanisms are otherwise coupled.

The response of *Giardia* trophozoites to changes in media tonicity has several potentially important implications. Giardiasis is a disease with a global distribution but a disproportionate impact on developing countries, contributing to malnutrition problems and to diarrheal illnesses that are particularly dangerous to young children and the immunocompromised. Current drug treatment methods exist, namely the administration of metronidazole, but they are not always effective or tolerated. Additionally, *Giardia* are capable of multiple drug resistance *in vitro* [7]; drug-resistant isolates have been found in patients although epidemics of resistant strains have not yet been reported [6]. As such, a simple, cost-effective, safe treatment that is easily administered and effective against all strains of the parasite would be particularly useful.

Our results demonstrate that sufficient changes in tonicity that are timed to take advantage of *Giardia*'*s* adaptation mechanisms can strongly and rapidly promote *Giardia* detachment. Since the tonicity changes leading to detachment in our experiments are within the range measured in the human small intestine [11], this method could be explored as a possible way to treat giardiasis or to enhance the effectiveness of present treatment methods. Ingestion of a low tonicity solution, such as water, followed by an interval that allows parasites to adjust to the lower lumenal tonicity, then followed by ingestion of a high tonicity solution, containing perhaps sucrose or PEG, may induce parasites to detach from the intestinal epithelium, as they did from human intestinal epithelial monolayers in our experiments (Figure 6, Video S1). Our experiments used a PEG/electrolyte mixture approved for use in humans as the medium base to support the feasibility of this treatment possibility. One central question facing application of this approach is whether sufficiently rapid swings in osmolarity can be achieved in the human intestine.

Our results provide clear evidence of *Giardia*'s sensitivity to and ability to adapt to the changing chemical environment of the host intestine. If tonicity-dependent detachment of *Giardia* is confirmed in human or animal model studies, our proposed “tonic shock therapy” may provide a useful strategy for enhancing the effectiveness of current treatments, helping to relieve symptoms in patients unable to take metronidazole or other antigiardial medications, or combating drug-resistant parasite strains.

## Supporting Information

Video S1. **Detachment of fluorescently labeled *Giardia* from C2_BBe_-1 monolayer under periodic tonicity swings of +/−150 mOsm/kg.**
Movie plays at 50X real time. Unattached cells were flushed out with growth medium (tonicity = 320 mOsm/kg) prior to the movie. The first few frames of the movie show the remainder of this growth medium flowing through the chamber. Cells are then exposed to hypertonic medium (470 mOsm/kg) for 8 minutes (3 minutes of flow at 0.3 mL/min, then 5 minutes static exposure). Cells do not detach during this time, but they become adjusted to the higher tonicity and are primed for detachment during the next 8 minutes of exposure to hypotonic medium (150 mOsm/kg). Media alternate between hyper- and hypotonic to the end of the movie, and additional cells detach with every change.
(8.82 MB MOV)

Video S2. **Detachment under tonic shock.**
*Giardia* cells are imaged in phase contrast at 100X in real time. The video shows cells attached to glass as they are exposed to hypotonic growth medium (196 mOsm/kg below normal growth medium). Two of the three cells detach under this change in tonicity. However, flagellar waveforms and cell morphology are not noticeably affected during detachment.
(3.44 MB MOV)

Figure S1 **Effect of medium tonicity on *Giardia* growth.**
(A) Cells grew normally in standard growth medium (300 mOsm/kg) in the 24-hour period after a three-minute exposure to hypotonic, isotonic, or hypertonic growth media. (B) Cells cultured for 48 hours in a range of media tonicities grew normally at values near 300 mOsm/kg. Cells grown in extremely hypo- and hyperosmotic media grew slowly or not at all over 24–48 hours of incubation.
(0.41 MB EPS)
